# Reduced tau expression in gastric cancer can identify candidates for successful Paclitaxel treatment

**DOI:** 10.1038/sj.bjc.6603182

**Published:** 2006-05-23

**Authors:** K Mimori, N Sadanaga, Y Yoshikawa, K Ishikawa, M Hashimoto, F Tanaka, A Sasaki, H Inoue, K Sugimachi, M Mori

**Affiliations:** 1 Department of Surgery, Kyushu University Hospital at Beppu, 4546 Tsurumihara, Beppu zip 874-0838, Japan; 2 Department of Surgery, Kyushu Central Hospital, Fukuoka, Japan; 3 Department of Pathology, Kyushu University Hospital at Beppu, Beppu, Japan; 4 Department of Pharmacology, Kyushu University Hospital at Beppu, Beppu, Japan

**Keywords:** tau, Paclitaxel, immunohistochemistry, susceptibility to chemotherapy

## Abstract

A recent study disclosed that breast cancer cases with low ‘tau’ expression can predict susceptibility to Paclitaxel administration. In the current study, the clinical significance of tau expression in gastric cancer cases was established by identifying candidates with Paclitaxel administration. Tissue specimens from 20 cases of in-operable or noncuratively resected gastric cancer were examined. Subsequent to the administration of 80 mg m^−2^ of Paclitaxel in six 3-h intravenous infusions, the clinical effectiveness of Paclitaxel was evaluated by the size of metastatic lesions with computed tomography. The status of the tau expression was determined by immunohistochemistry. Based on a previously reported classification scheme, six were classified as tau-negative expression (0, 1+) cases and 14 were classified as tau-positive expression (2+, 3+) cases. All six (100%) cases of tau-negative expression showed a favourable response (partial response or minor response) to Paclitaxel administration. However, 12 (86%) of the 14 cases of tau-positive expression showed progressive disease (*n*=11) or no change (*n*=1) after Paclitaxel administration. The serum carcinoembryonic antigen values of the six cases of tau-negative expression were markedly decreased in comparison to the 14 tau-positive cases. These data indicate that tau-negative expression can be used to select gastric cancer patients, which will favourably respond to Paclitaxel treatment.

It has been more than 10 years since Paclitaxel emerged as a successful drug for the treatment of advanced gastric cancer. However, the overall response rate to this drug remains low. The solitary administration of Paclitaxel is insufficient for clinical benefit in comparison to combination treatments with other drugs, such as anthracycline, 5-fluorouracil (FU), and irinotecan (Park *et al*, 2004). In fact, combination therapy with anthracycline and Paclitaxel improved disease-free survival (Henderson *et al*, 2003). On the other hand, a Phase I study of Paclitaxel revealed a high incidence of alopecia or grade 1–2 leukocytopenia (Cascinu *et al*, 1997). Therefore, if appropriate candidates for Paclitaxel administration can be identified at the time of diagnosis, a high antitumour effect and reduced side effects may improve the therapeutic index of the drug.

Recently, Rouzier *et al* reported that tissue arrays from 122 independent but similarly treated breast cancer patients were used for validation by immunohistochemistry (IHC). In total, 74% of pathologically complete response (pCR) cases were tau protein negative. In multivariate analysis, nuclear grade (*P*<0.01), age <50 (*P*=0.03), and tau-negative status (*P*=0.04) were independent predictors of pCR. The authors also showed decreased Paclitaxel binding and reduced microtubule polymerisation in breast cancer cells after preincubation of tubulin with tau protein (Rouzier *et al*, 2005).

The purpose of this study was to clarify the significance of tau expression in gastric cancer from the view point of Paclitaxel administration.

## PATIENTS AND METHODS

### Patients and samples

A total of 20 cases of advanced and nonresectable gastric cancer with Paclitaxel administration were used for this study. The average age at treatment was 66 years, ranging from 52 to 81 years of age. There were 14 male and six female patients. In all, 14 patients were treated at Kyushu University Hospital at Beppu, and six were treated at Kyushu Central Hospital. Gastric cancer tissues and the corresponding normal tissues were obtained from nine cases of palliative surgery (noncurative operations). Biopsy specimens were obtained from eight recurrent cases and three inoperable cases. Recurrent disease was recognised in the liver, lungs, abdominal lymph nodes, and peritoneal dissemination. Histological findings were as follows: nine cases of differentiated type (two well-differentiated adenocarcinomas and seven moderately differentiated adenocarcinomas); nine of the undifferentiated type (five poorly differentiated adenocarcinomas and four signet-ring cell carcinomas); and two cases of mucinous adenocarcinoma (Table 1).

### Chemotherapy regimens and evaluations

Paclitaxel was administered as the first-line chemotherapy in 10 (50%) of the 20 cases, and there was no deviation of the first-line-treated number of cases between the six cases of tau-negative expression and the 14 cases of tau-positive expression. The remaining 10 cases were treated by 5-FU plus cisplatin followed by Paclitaxel as the second-line chemotherapy. A 3-h intravenous infusion of Paclitaxel (80 mg m^−2^) was given on days 1, 8, and 15, and was repeated twice every 4 weeks (Yeh *et al*, 2005). Tumour response was evaluated by measuring the serum level of carcinoembryonic antigen (CEA) and the size of the metastatic lesions with computed tomography (CT) 2 months after the initial administration of Paclitaxel. Responses were defined as complete (CR, no clinical/radiographic detectable disease), partial (PR, >/=50% reduction in disease), minor (MR, <50% reduction), no change (NC), or progressive disease (PD).

### IHC

Sections (4 *μ*m) were prepared for tissue slides. Antigen retrieval was performed by boiling in citrate buffer (pH 6.0) for 10 min in a microwave oven after deparaffinisation. Nonspecific binding was blocked by incubating sections in ^*^PBS containing 1% normal bovine serum and 0.3% Triton-X 100. The slides were rinsed three times with PBS for 3 min and incubated with an anti-TAU-1 monoclonal antibody (5 *μ*g ml^−1^; clone #PC1C6) (Cedarlane, Hornby, Ontario, Canada) diluted in PBS containing 1% normal bovine serum (Binder *et al*, 1985). The slides were again washed with PBS, changing the solution three times over a 3-min period, after which detection was performed with a standard secondary antibody system (Taylor, 1978; Hsu *et al*, 1981; Falini and Taylor, 1983). Sections of normal breast were used as external reference control for staining and scoring. The previous reported IHC scoring was followed: 0, no staining; 1+, less staining than normal epithelium; 2+, similar to normal epithelium; 3+, uniform staining more intense than normal cells. In accord with the previous study, cases with 0 or 1+ staining intensity were considered tau negative, and tumours with 2+/3+ staining were considered tau positive (Rouzier *et al*, 2005).

### Statistical analysis

Each clinicopathological variable was compared between the tau-positive and -negative expression groups, and evaluated with Fisher's exact test.

## RESULTS

**Figure 1:**
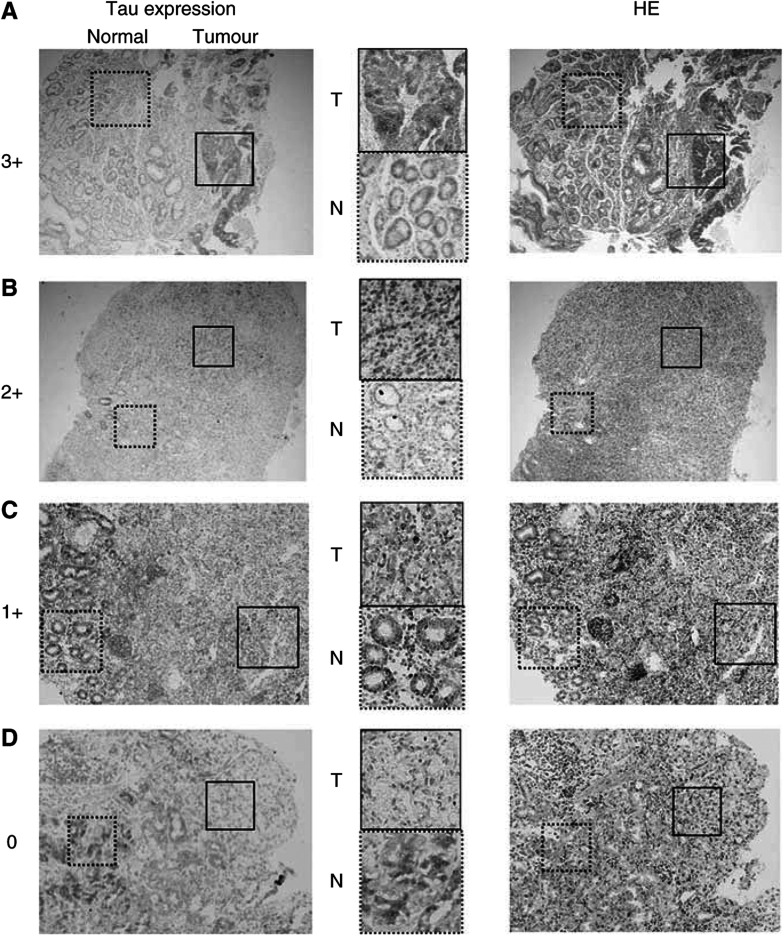
Representative Tau expression pattern in gastric cancer cases for the IHC score (3+, 2+, 1+ and 0), whereas the right-hand side indicates the haematoxylin eosin staining (× 40 magnification). (**A**) 3+: There is strong granular staining in the cytoplasm of cancer cells obtained from biopsy specimens, moderately differentiated adenocarcinoma. This case was inoperable owing to the pleural effusions and the mediastinum lymph node metastases; a Paclitaxel nonresponsive case (PD). (**B**) 2+: Both nuclei and cytoplasm of the cancer cells are strongly positive. Normal mucosal glands are also positive. This case originated from a resected specimen of signet ring cell carcinoma after distal gastrectomy. A noncurative or a palliative operation was performed because of the dissemination of cancer cells into the retroperitoneum; a Paclitaxel nonresponsive case (PD). (**C**) 1+: A signet ring cell carcinoma case with positive tau expression is observed both in normal and tumour tissues. However, normal cells have a stronger signal than those of the tumorous tissue. No remarkable change was observed after Paclitaxel administration (NC). (**D**) 0: While the normal gastric glands (right) are strongly stained, the signet-ring type cancer cells (left) are completely negative. This case was partially responsive case (PR).

As shown in Figure 1, according to the tau protein expression pattern in the cancerous and corresponding normal tissues, two, 12, four, and two cases showed 0, 1+, 2+, and 3+ staining, respectively. The clinicopathological variables between 14 tau-positive cases (3+, 2+) and six tau-negative cases (0, 1+) are summarized in Table 1. There was no significant difference between the two groups with respect to sex, histology, or pretreatment status. However, there was a significant difference between the clinical effectiveness of Paclitaxel and tau expression status. All six (100%) cases of tau-negative expression responded to Paclitaxel administration (PR, MR), but 12 (86%) of the 14 tau-positive cases showed no response (PD, NC) to Paclitaxel.

In addition, the serum CEA value was examined as a comparison of the clinical effectiveness between positive and negative tau expression. The difference did not reach statistical significance; however, the serum CEA value decreased in five (83%) of six tau-negative cases after Paclitaxel administration. On the other hand, it was increased in 10 (71%) of 14 tau-positive cases after Paclitaxel administration. As for the alteration of the practical CEA value after Paclitaxel administration, the six patients with tau-negative expression reduced the value to 98% of the pretreatment value, whereas it was increased by 129% in the 14 tau-positive patients.

Computed tomography evaluation of the six tau-negative cases revealed that two cases showed a reduction in tumour size in the minor curvature of the stomach (MR), one case exhibited ascites elimination (PR), and the remaining three cases had a reduction in the size of paraaortic lymph node swelling (one PR and two MR) with the administration of Paclitaxel.

In addition, adverse events resulting from the administration of Paclitaxel were investigated. Leukocytopenia defined as grade 2 or 3 (500 mm^−3^<neutrophil<1500 mm^−3^) was observed in five (83%) out of six cases of tau-negative expression and in 10 (71%) out of 14 cases of tau-positive expression, with no significant difference between the groups. There was also no significant difference between the groups regarding the occurrence of alopecia, which was recognized in four (67%) out of six of those with tau-negative expression and eight (57%) out of 14 cases with tau-positive expression.

Furthermore, a comparison of the improvement of symptoms after Paclitaxel administration in tau-negative cases and tau-positive cases was as follows: vanishing ascites were observed in four (67%) out of six tau-negative cases and four (29%) out of 14 tau-positive cases. In addition, appetite loss decreased in three (50%) out of six and three (21%) out of 14 cases of tau positive.

## DISCUSSION

Various molecules have been reported as markers for prediction of a high level of sensitivity to Paclitaxel. Vikhanskaya *et al* (1998) reported that the inactivation of p53 is susceptible to Paclitaxel in ovarian cancer (Rouzier *et al*, 2005), and Reinecke *et al* (2000a, 2000b) described the expression and function of P-glycoprotein in renal cell carcinoma. Moreover, the overexpression of antiapoptotic genes (Wang *et al*, 2005), the upregulation of cyclin A (Takahashi *et al*, 2005), the overexpression of oestrogen receptor (Dougherty *et al*, 2004), and the reduced expression of tau (Rouzier *et al*, 2005) have all been reported as Paclitaxel susceptibility markers in breast cancer. As for colorectal cancers, the expression of MDR-1, bcl-2, and bax each contribute to Paclitaxel sensitivity (Sharma *et al*, 2000).

In the current study, we focus on tau expression as the susceptible marker for Paclitaxel against gastric cancer, because, there have been no definitive studies of Paclitaxel sensitivity markers in gastric cancer. In logical consideration, we expect that tau can be a suitable powerful marker for the microtubulising agent, Paclitaxel. Because tau binds to microtubules strongly without Paclitaxel, whereas microtubules assembled in the presence of Paclitaxel show moderate binding affinity and rapid dissociation kinetics of tau protein (Rouzier *et al*, 2005).

Consistent with the report of breast cancer, we found that gastric cancer cases with low levels of tau expression also indicate a higher susceptibility to Paclitaxel treatment. All six (100%) cases of low tau expression not only had a favourable clinical response but also showed an improvement of clinical symptoms, such as ascites and appetite loss due to Paclitaxel administration. Only two (14.3%) of the 14 cases with high tau expression showed a similar response. However, in spite of the improved effectiveness, side effects such as bone marrow suppression, alopecia, and appetite loss (recorded within 2 days after Paclitaxel administration) were observed equally between patients of low and high tau expression. This observation indicates that tau expression status cannot become an indicator of adverse effects due to Paclitaxel. Therefore, other indicators, such as single-nucleotide polymorphisms in the genomic region, must be used to predict which patients are at high risk for severe adverse effects. Studies are ongoing in an effort to find these markers.

As Rouzier *et al* also described, low tau expression renders microtubules more vulnerable to Paclitaxel and makes breast cancer cells hypersensitive to this drug. They postulated that tau could be a molecular target therapy to elevate sensitivity to Paclitaxel by the inhibition of tau expression in breast cancer cases (Rouzier *et al*, 2005). We propose that tau inhibition would also improve the clinical effectiveness of Paclitaxel in gastric cancer cases.

In conclusion, low tau expression denotes a higher susceptibility to Paclitaxel treatment in gastric cancer. This finding suggests that tau expression could be used at diagnosis to select patients for whom Paclitaxel treatment would be most beneficial. However, many more cases will have to be examined before tau expression can be applied to practical clinical treatment.

## Figures and Tables

**Table 1:**
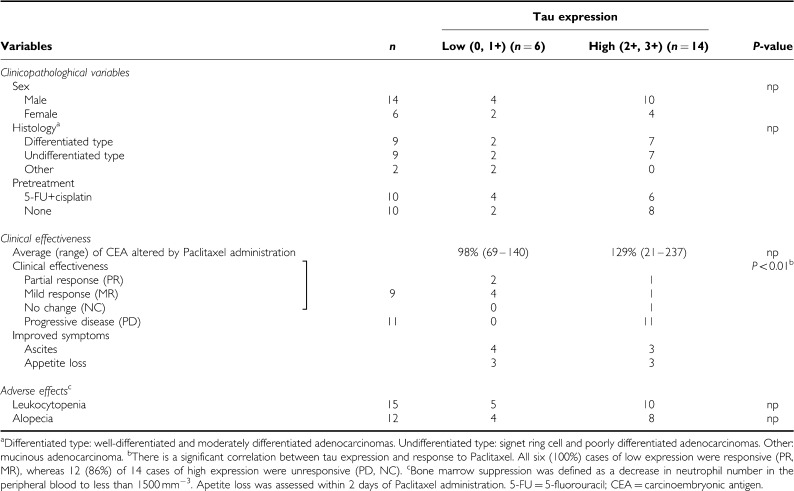
Relationship between tau expression and the clinical usefulness of paclitaxel in 20 paclitaxel-administrated cases of gastric cancer

## References

[bib1] Binder LI, Frankfurter A, Rebhun LI (1985) The distribution of tau in the mammalian central nervous system. J Cell Biol 101: 1371–1378 3930508 10.1083/jcb.101.4.1371 PMC2113928

[bib2] Cascinu S, Ficarelli R, Safi MA, Graziano F, Catalano G, Cellerino R (1997) A phase I study of paclitaxel and 5-fluorouracil in advanced gastric cancer. Eur J Cancer 33: 1699–1702 9389936 10.1016/s0959-8049(97)00134-2

[bib3] Dougherty MK, Schumaker LM, Jordan VC, Welshons WV, Curran EM, Ellis MJ, El-Ashry D (2004) Estrogen receptor expression and sensitivity to paclitaxel in breast cancer. Cancer Biol Ther 3: 460–467 15020841 10.4161/cbt.3.5.810

[bib4] Falini B, Taylor CR (1983) New developments in immunoperoxidase techniques and their application. Arch Pathol Lab Med 107: 105–117 6187311

[bib5] Henderson IC, Berry DA, Demetri GD, Cirrincione CT, Goldstein LJ, Martino S, Ingle JN, Cooper MR, Hayes DF, Tkaczuk KH, Fleming G, Holland JF, Duggan DB, Carpenter JT, Frei III E, Schilsky RL, Wood WC, Muss HB, Norton L (2003) Improved outcomes from adding sequential Paclitaxel but not from escalating Doxorubicin dose in an adjuvant chemotherapy regimen for patients with node-positive primary breast cancer. J Clin Oncol 21: 976–983 12637460 10.1200/JCO.2003.02.063

[bib6] Hsu SM, Raine L, Fanger H (1981) A comparative study of the peroxidase–antiperoxidase method and an avidin–biotin complex method for studying polypeptide hormones with radioimmunoassay antibodies. Am J Clin Pathol 75: 734–738 6165237 10.1093/ajcp/75.5.734

[bib7] Park SR, Oh DY, Kim DW, Kim TY, Heo DS, Bang YJ, Kim NK, Kang WK, Kim HT, Im SA, Suh JH, Kim HK, Kim HK (2004) A multi-center, late phase II clinical trial of Genexol (paclitaxel) and cisplatin for patients with advanced gastric cancer. Oncol Rep 12: 1059–1064 15492793

[bib8] Reinecke P, Knopf C, Schmitz M, Schneider EM, Gabbert HE, Gerharz CD (2000a) Growth inhibitory effects of paclitaxel on human epithelioid sarcoma *in vitro*: heterogeneity of response and the multidrug resistance phenotype. Cancer 88: 1614–1622 10738220 10.1002/(sici)1097-0142(20000401)88:7<1614::aid-cncr16>3.0.co;2-x

[bib9] Reinecke P, Schmitz M, Schneider EM, Gabbert HE, Gerharz CD (2000b) Multidrug resistance phenotype and paclitaxel (Taxol) sensitivity in human renal carcinoma cell lines of different histologic types. Cancer Invest 18: 614–625 11036469 10.3109/07357900009032828

[bib10] Rouzier R, Rajan R, Wagner P, Hess KR, Gold DL, Stec J, Ayers M, Ross JS, Zhang P, Buchholz TA, Kuerer H, Green M, Arun B, Hortobagyi GN, Symmans WF, Pusztai L (2005) Microtubule-associated protein tau: a marker of paclitaxel sensitivity in breast cancer. Proc Natl Acad Sci USA 102: 8315–8320 15914550 10.1073/pnas.0408974102 PMC1149405

[bib11] Sharma N, Ramachandran S, Bowers M, Yegappan M, Brown R, Aziz S, Chapman R, Yu BW (2000) Multiple factors other than p53 influence colon cancer sensitivity to paclitaxel. Cancer Chemother Pharmacol 46: 329–337 11052631 10.1007/s002800000155

[bib12] Takahashi T, Yamasaki F, Sudo T, Itamochi H, Adachi S, Tamamori-Adachi M, Ueno NT (2005) Cyclin A-associated kinase activity is needed for paclitaxel sensitivity. Mol Cancer Ther 4: 1039–1046 16020661 10.1158/1535-7163.MCT-04-0282

[bib13] Taylor CR (1978) Immunoperoxidase techniques: practical and theoretical aspects. Arch Pathol Lab Med 102: 113–121 76464

[bib14] Vikhanskaya F, Vignati S, Beccaglia P, Ottoboni C, Russo P, D'Incalci M, Broggini M (1998) Inactivation of p53 in a human ovarian cancer cell line increases the sensitivity to paclitaxel by inducing G2/M arrest and apoptosis. Exp Cell Res 241: 96–101 9633517 10.1006/excr.1998.4018

[bib15] Wang Q, Tsao SW, Ooka T, Nicholls JM, Cheung HW, Fu S, Wong YC, Wang X (2005) Anti-apoptotic role of BARF1 in gastric cancer cells. Cancer Lett (Epub ahead of print) 10.1016/j.canlet.2005.06.023 16054293

[bib16] Yeh KH, Lu YS, Hsu CH, Lin JF, Hsu C, Kuo SH, Li SJ, Cheng AL (2005) Phase II Study of weekly Paclitaxel and 24-H infusion of high-dose 5-fluorouracil and leucovorin in the treatment of recurrent or metastatic gastric cancer. Oncology 69: 88–95 16088236 10.1159/000087304

